# Dentine Tubule Occlusion by Novel Bioactive Glass-Based Toothpastes

**DOI:** 10.1155/2018/5701638

**Published:** 2018-04-04

**Authors:** Luiza Pereira Dias da Cruz, Robert G. Hill, Xiaojing Chen, David G. Gillam

**Affiliations:** Oral Bioengineering, Barts and the London School of Medicine and Dentistry, QMUL, London, UK

## Abstract

There are numerous over-the-counter (OTC) and professionally applied (in-office) products and techniques currently available for the treatment of dentine hypersensitivity (DH), but more recently, the use of bioactive glasses in toothpaste formulations have been advocated as a possible solution to managing DH. *Aim*. The aim of the present study, therefore, was to compare several bioactive glass formulations to investigate their effectiveness in an established in vitro model. *Materials and Methods*. A 45S5 glass was synthesized in the laboratory together with several other glass formulations: (1) a mixed glass (fluoride and chloride), (2) BioMinF, (3) a chloride glass, and (4) an amorphous chloride glass. The glass powders were formulated into five different toothpaste formulations. Dentine discs were sectioned from extracted human teeth and prepared for the investigation by removing the cutting debris (smear layer) following sectioning using a 6% citric acid solution for 2 minutes. Each disc was halved to provide test and control halves for comparison following the brushing of the five toothpaste formulations onto the test halves for each toothpaste group. Following the toothpaste application, the test discs were immersed in either artificial saliva or exposed to an acid challenge. *Results*. The dentine samples were analyzed using scanning electron microscopy (SEM), and observation of the SEM images indicated that there was good surface coverage following artificial saliva immersion. Furthermore, although the acid challenge removed the hydroxyapatite layer on the dentine surface for most of the samples, except for the amorphous chloride glass, there was evidence of tubular occlusion in the dentine tubules. *Conclusions*. The conclusions from the study would suggest that the inclusion of bioactive glass into a toothpaste formulation may be an effective approach to treat DH.

## 1. Introduction

### 1.1. Overview

Dentine hypersensitivity (DH) affects approximately 10–30% of the adult population and may have a direct impact on the individual's quality of life [1]. There are numerous over-the-counter (OTC) and professionally applied (in-office) products and techniques available for the treatment of DH. Recently, the use of bioactive glasses in toothpaste formulations has been advocated as a possible long-term solution for managing DH [2]. Currently, most of the research activity focusses on the hydrodynamic theory as the basis for the therapeutic treatment of DH. The rationale being that, by blocking the dentinal tubules (tubular occlusion), there will a corresponding reduction of the fluid flow through dentine (dentine permeability) and a subsequent relief of pain [1, 3]. The mechanisms underpinning the hydrodynamic theory are generally investigated in several recognized models, for example, in vitro, in situ, in vivo human studies and animal studies (for nerve desensitizing mechanisms). The aim of the present in vitro study, therefore, was to investigate the effectiveness of experimental bioactive glasses designed for toothpaste formulations.

An ideal desensitizing agent should have a rapid action with long-term effects, be non-irritant to pulp, painless, easy to apply, and should not stain the tooth [4]. Toothpastes are considered the most economic method for using desensitizing in-home treatments and generally are classified by the regulatory authorities on the ingredients within the formulation (e.g., cosmetic and medicine/drug) [5, 6]. There are a plethora of products that are being developed for this condition, but currently, there does not appear to be one ideal product that can completely resolve the problem.

Bioactive materials have been considered for both medical and dental use particularly in bone defects. For example, in 1969, bioactive glasses were discovered as a second-generation alternative for the bonding of bone to an implant within the host's tissue through a chemical reaction. One of the advantages of these materials was that they have the capacity of producing hydroxyapatite and induce osteogenesis in physiological systems [7]. Since 1985, 45S5 Bioglass has been used clinically, as a third generation of biomaterials, for tissue reparation using gene activation properties. The first Bioglass-containing material established in the market was initially used to treat conductive hearing loss and also in several head and neck surgeries [7]. In dentistry, the application of bioactive glasses was related to bone replacement implants in edentulous patients, to provide a more stable ridge for denture construction. It was also used for periodontal diseases (PerioGlas®) and bone defects as a method of reconstruction of the bone. Dentine and bone have similarities in terms of tissue composition (e.g., hydroxyapatite); hence, biocompatible glasses may be an efficient material for incorporating into toothpaste formulations as a tubular occludent [8–10]. The composition of bioactive glasses is silicon, sodium, calcium, and phosphorus oxides with specific percentages. In addition, fluoride can be incorporated into the glass, and both fluoride and calcium ions are released in the presence of saliva [9, 10]. When in contact with a biological fluid such as saliva, Bioglass particles react and three processes occur: (1) leaching and formation of silanols, (2) dissolution of the glass network, and (3) precipitation. Precipitation is an important process for occluding dentine tubules. The formation of a layer composed of calcium and phosphate induced by the release of these ions from the glass can mechanically occlude dentinal tubules and lower fluid flow within the dentine. This layer is crystallized into hydroxyapatite, and the presence of silica can accelerate the maturation of hydroxyapatite. In the bone, bioactive glass triggers an osteoblast cell cycle leading to rapid cell proliferation and differentiation [7, 9, 11, 12].

Toothpaste formulations containing potassium designed to treat sensitive teeth can also have an analgesic effect. For example, potassium saline is responsible for maintaining high levels of potassium ion extracellularly, preventing repolarization of the nerve cell membrane and inhibiting the transmission of impulses. In brief, potassium nitrate has been postulated to act by blocking neural transmission to reduce DH symptoms. However, there are limited clinical data in humans to support the mode of action of potassium ions reducing DH. Several studies have reported that toothpastes containing calcium sodium phosphosilicate or NovaMin® (GSK) can occlude the dentinal tubules more effectively than potassium salts as evidenced in clinical studies and in vitro studies immersing the products in artificial saliva [6, 11, 12]. Usually toothpaste formulations are based on fluoride (to protect against caries), an abrasive component that provides the cleaning ability, substances that inhibit bacterial growth, and other ingredients [6, 12, 13]. Fluoride is a compound that may aid remineralization in enamel although evidence regarding its effect on DH is limited. It has been demonstrated, however, that fluoride in toothpastes can create a precipitation onto the dentine surface and block the dentine tubules, increasing resistance against an acidic challenge [6]. The precipitation however may also contain silica particles that can occlude the dentinal tubules rather than the fluoride ion per se. The suggested actions of a bioactive glass toothpaste were through its chemical ability to occlude dentine tubules by the formation of calcium-phosphorous precipitates, calcium fluoride, and fluorapatite. Fluoride in toothpaste formulations however is important for its role in caries prevention by reducing the rate of demineralization, promoting remineralization of damaged tissue, and decreasing acid production by interfering with oral bacteria in the tooth biofilm, functioning as a biocide against *S. mutans.* Nevertheless, fluoride in combination with other ions may also enhance the effectiveness of any desensitizing effects [10, 14]. Toothpaste formulations may also induce the formation of calcium, phosphate, and fluoride and contribute to intratubular mineralization [6, 12, 13]. In addition, the use of calcium phosphate products has been considered promising for the treatment of DH. Calcium is also important for the remineralization in tooth restorations as it was the primary component of hydroxyapatite, with apatite as a form of calcium phosphate, which can be used in dental materials (e.g., hydroxyapatite and fluorapatite).

Precipitation of hydroxyapatite onto the exposed dentine surface can occlude dentine tubules, and it has been shown that calcium phosphate may occlude dentinal tubules without inhibiting the spontaneous remineralization of the tooth surface [10, 15]. Moreover, toothpaste formulations can also contain strontium, stannous, and calcium phosphate which can form physical barriers that may occlude the dentine tubules. These mechanisms occur by precipitating insoluble metal compounds on the dentine surface. Stannous chloride has also been reported to be effective in occluding dentine tubules although NovaMin® has been reported to be more effective than a strontium chloride and placebo toothpaste, particularly when exposed to citric acid and artificial saliva. [16] Stannous fluoride may also block the dentine tubules by forming SnF_2_ and CaF_2_. Additionally, strontium chloride has been reported to block the dentine tubules [6]. Tubule occlusion may, however, occur naturally through the normal remineralization processes by saliva and by dentine sclerosis through secondary dentine formation. Saliva also has a protective function against tooth wear. The biofilm layer has been reported to promote remineralization and reduce any mineral loss [14]. Therefore, using some of these dental products may help protect the dentine in order to enhance its resistance to both mechanical and chemical attack. One method of increasing the dentine surface resistance to wear by acid erosion and abrasion is to increase its mineral density; alternatively occluding the dentine tubules with a mineral substance, such as a calcium and phosphate toothpaste, would increase acid resistance of the dentine [13, 16].

### 1.2. Aim of the Project

The aim of the project, therefore, was to compare different bioactive glass formulations to investigate their effectiveness in the in vitro environment.

## 2. Materials and Methods

A 45S5 glass was synthesized in the laboratory together with several other glass formulations and subsequently formulated into five different toothpaste formulations (Section 2.4).

### 2.1. Preparation of Samples

Caries-free extracted mandibular and maxillary molars were collected from the tooth bank with approval from the Queen Mary Research Ethics Committee QMREC 2011/99.

The teeth were cleaned with deionized water and stored in 70% ethanol. Each tooth was sliced into mid coronal sections by a diamond cut-off wheel machine. The teeth were embedded in an impression material to make blocks to stabilize them. The sections were required to be less than 1 mm thick; therefore, the machine was set to approximately 0.600 mm thickness. Subsequently, the dentine discs were polished using a carbide abrasive paper of P800, P2500, and P4000 consequently. A micrometre was used to measure the thickness after polishing, and it was established to be approximately 0.3 mm.

### 2.2. Glass Manufacture

The 45S5 bioactive glass was manufactured in the laboratory within the University Department. The reagents including SiO_2_ (45%), CaO (24.5%), P_2_O_5_ (6.0%), and Na_2_O (24.5%) were mixed and placed in a crucible and melted at 1390°C for 1 hour in an electric furnace. The mixture was then quenched into cold water to prevent crystallization and ground in a Gyro Mill for two sets of 7 minutes. Finally, the particles were separated into two groups: (1) ≥38 microns and (2) ≤38 microns using a 38 *μ*m sieve. The glass with a particle size ≤38 microns was used for further characterization.

### 2.3. Artificial Saliva and Toothpaste Application

The artificial saliva was formulated using potassium chloride (2.236 g/L), potassium dihydrogen phosphate (1.361 g/L), sodium chloride (0.759 g/L), calcium chloride dihydrate (0.441 g/L), mucin (2.200 g), and sodium azide (0.2 g). These reagents were weighed in an electronic balance and dissolved in 8000 mL of deionized water; the pH was adjusted to 6.5 with KOH.

The paste was manufactured following the protocol by Mahmood et al. [12]. The components included glycerol, Carbopol (polyacrylic acid), PEG400 (polyethylene glycol), Syloid 63, synthetic amorphous silica, K acesulfame, titanium dioxide, and Na lauryl S (sodium dodecyl sulfate). The ingredients were weighed and mixed thoroughly in a plastic container. The pastes were divided into five portions with approximately 17 g each. Five batches of bioactive glass containing toothpastes (laboratory-manufactured 45S5, mixed glass, commercial BioMinF, CDL chloride glass, and amorphous chloride glass) were formulated by adding 1 g of bioactive glass into the paste and mixed thoroughly.

### 2.4. Experimental Design

Five different types of bioactive glasses were investigated. The laboratory-manufactured 45S5, mixed glass containing fluoride and chloride, commercial BioMinF (BioMin Technologies Ltd., London, UK), chloride glass, and amorphous chloride glass (Table 1).

### 2.5. Scanning Electron Microscopy Study

The discs were immersed in 6% citric acid for 30 seconds to remove the smear layer. The etched discs were then rinsed with deionized water and dried prior to the commencement of the experiment. For each glass-based toothpaste, one disc was cut into four halves. The first half was the control without brushing, and the other halves were brushed with a fixed amount of 50 mg toothpaste for 30 seconds. The second half was rinsed with deionized water, dried, and stored for SEM analysis. The third half was, subsequently to brushing, immersed in artificial saliva for 1 hour and then dried and stored for SEM. The fourth half was immersed in 6% citric acid for 2 minutes, rinsed with deionized water, dried, and stored for SEM (Table 2).

### 2.6. Quantification of Tubule Occlusion Based on SEM Observation

The number of tubules was assumed to be constant between the control section of the etched dentine and the treated dentine section. This assumption was made because in many cases, the individual tubules were no longer visible after treatment. The number of tubules in each section of the control etched dentine varied between 56 and 72. However, for each sample, the control etched section was taken from the same mid coronal dentine slice and in close proximity to the etched dentine. Tubules were classified as fully open, partially occluded where particles were observed within the tubule or where materials were observed to have reduced the diameter significantly compared to the control section, or fully occluded. Since fully occluded tubules in many cases could not be observed, the number of fully occluded tubules was assumed to be the number of tubules in the control section minus the number of open tubules and partially occluded tubules. The results were converted to a percentage. Typically about 100 tubules were examined for each treatment representing a random statistical error of about 10%.

The dentine discs were mounted onto stubs with conducting carbon cement. Each part of the disc was mounted flat with the upper side exposed for brushing. The samples were subsequently sputter coated with gold/palladium for SEM analysis. The images were obtained from different fields of the disc at alternative magnifications such as 5000x and 10000x.

## 3. Results

It was evident from previous research in the department that the bioactive glasses have the capacity of occluding dentine tubules and as such may be an effective treatment for DH. In the present investigation, we used laboratory-made artificial saliva to mimic the in vivo environment following brushing of the discs. The results showed very good surface deposition with characteristic plate-like crystals or needle crystal-like formations in the various toothpaste samples. In the 45S5 sample, there was evidence that the Bioglass did not form apatite directly but may have formed a phase called octacalcium phosphate (OCP) which displays a plate-like morphology which may, then convert to a hydroxycarbonated apatite. The use of artificial saliva immersion following the toothpaste application was a useful addition to the methodology normally employed in in vitro studies in that it provided a similar environment to the oral cavity. For example, in the SEM images, there was clear evidence for hydroxyapatite formation with several hexagonal crystals observed following artificial saliva immersion. Apatite often has a hexagonal structure that can be recognized in the SEM images. Moreover, hydroxyapatite is a more stable crystal compared to other calcium phosphates, under physiological conditions. However, when exposed to an acid challenge, most of the hydroxyapatite layer formed with the artificial saliva dissolved although there was still a degree of tubule occlusion present.

**Figure 1:**
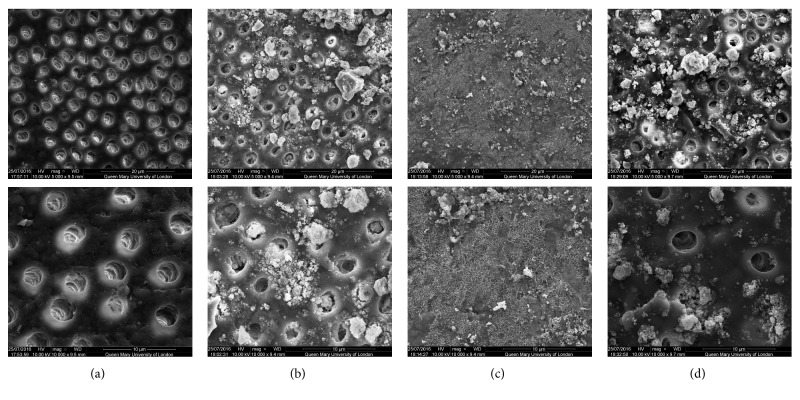
SEM images of the dentine surface morphology in disc 1 treated with a 45S5 glass-based toothpaste at 5000x (top) and 10000x (bottom) magnifications. (a) Control; (b) after brushing with a bioactive glass-based toothpaste; (c) after brushing with a bioactive glass-based toothpaste + artificial saliva immersion for 1 hour; (d) after brushing with a bioactive glass-based toothpaste + acid challenge with 6% citric acid.

**Figure 2:**
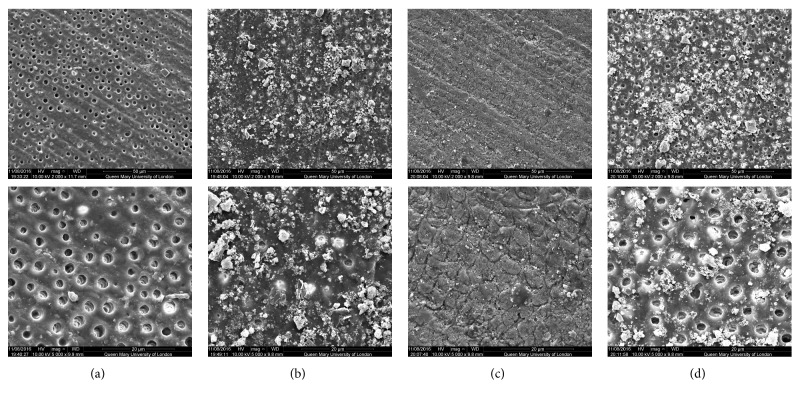
SEM images of the dentine surface morphology in disc 2 treated with a mixed glass-based toothpaste at 2000x (top) and 5000x (bottom) magnifications. (a) Control; (b) after brushing with a bioactive glass-based toothpaste; (c) after brushing with a bioactive glass-based toothpaste + artificial saliva immersion for 1 hour; (d) after brushing with a bioactive glass-based toothpaste + acid challenge with 6% citric acid.

**Figure 3:**
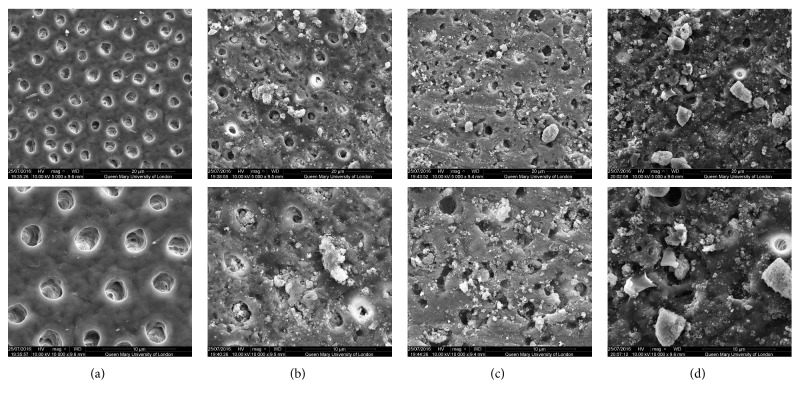
SEM images of the dentine surface morphology in disc 3 treated with a BioMinF glass-based toothpaste at 5000x (top) and 10000x (bottom) magnifications. (a) Control; (b) after brushing with a bioactive glass-based toothpaste; (c) after brushing with a bioactive glass-based toothpaste + artificial saliva immersion for 1 hour; (d) after brushing with a bioactive glass-based toothpaste + acid challenge with 6% citric acid.

**Figure 4:**
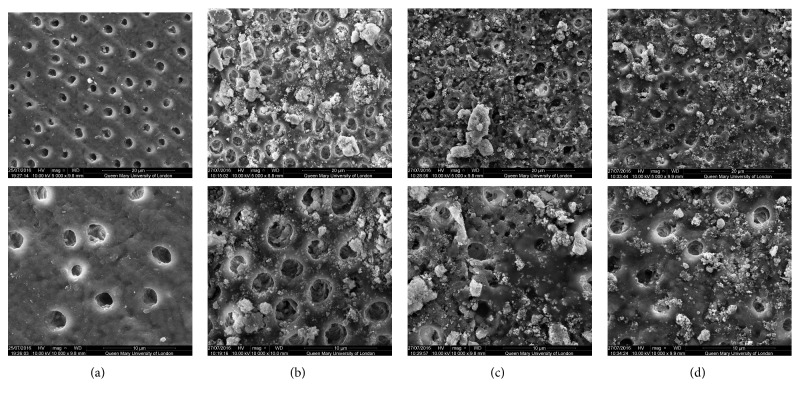
SEM images of the dentine surface morphology in disc 4 treated with a chloride glass-based toothpaste at 5000x (top) and 10000x (bottom) magnifications. (a) Control; (b) after brushing with a bioactive glass-based toothpaste; (c) after brushing with a bioactive glass-based toothpaste + artificial saliva immersion for 1 hour; (d) after brushing with a bioactive glass-based toothpaste + acid challenge with 6% citric acid.

**Figure 5:**
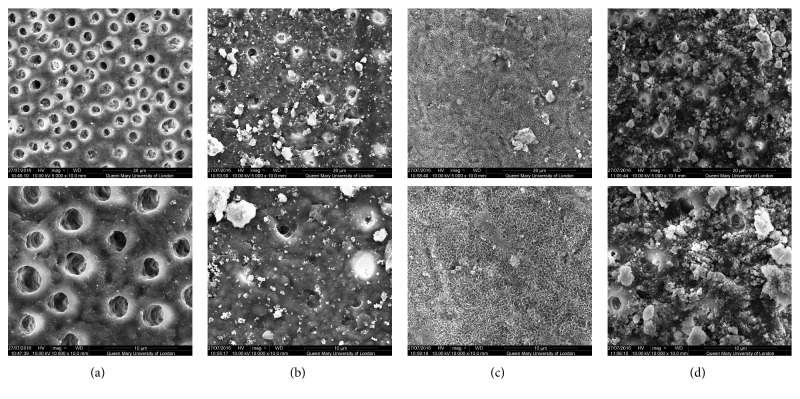
SEM images of the dentine surface morphology in disc 5 treated with an amorphous chloride glass-based toothpaste at 5000x (top) and 10000x (bottom) magnifications. (a) Control; (b) after brushing with a bioactive glass-based toothpaste; (c) after brushing with a bioactive glass-based toothpaste + artificial saliva immersion for 1 hour; (d) after brushing with a bioactive glass-based toothpaste + acid challenge with 6% citric acid.

SEM analysis of the test and control discs can be observed in the following figures (Figures 1–5); for example, an SEM image of a control etched dentine surface with visible open tubules can be observed in Figure 1. Please note that a residual smear layer deposit was present due to the samples not being cleaned ultrasonically before the commencement of the study in order to mimic real-life conditions.

The SEM images for the toothpaste containing 45S5 can be observed in Figure 1(a). In Figure 1(b), it may be noticed that, after brushing, some of the tubules were occluded by the particles. However, in Figure 1(c), when immersed in artificial saliva for 1 hour, all the tubules were occluded, as indicated by the clear formation of a layer covering the treated surface. This formation would lend some support to the fact that the effects of the product were improved when immersed in artificial saliva compared to when the discs were only brushed without immersion in saliva. After the acid challenge, in Figure 1(d), a significant part of that layer covering the tubules was removed, although it can be noted that some of the tubules were still occluded.

Figure 2(b) showed an improved coverage of the mixed glass product compared to 45S5. Nevertheless, the layer formed with the artificial saliva immersion was similar but with different formations of particles (Figure 2(c)). Following the acid challenge, as Figure 2(d) revealed, the layer was again rinsed away although some of the tubules remained occluded.

Figure 3(b) showed good coverage of the dentine surface with BioMinF. However, when immersed in artificial saliva, the layer formed was different to that formed with 45S5 and the mixed glass (Figure 3(c)). Particles of various sizes can be observed on the dentine surface and within the tubule lumen. Following the acid challenge, the results showed improvement compared to the other glasses. BioMinF was not totally washed away, and Figure 3(d) would suggest that almost the same amount of the dentine tubules maintained their occlusion (Figure 3(b)).

In Figure 4, the chloride glass sample showed basically the same coverage as with the other figures. This observation would suggest that although the layer covering the tubules was not formed with artificial saliva, the particles were not dissolved away with the acid challenge. Nevertheless, some of the tubules still remained open.

Finally, Figure 5 showed an excellent surface coverage with the amorphous chloride glass-based toothpaste. Particles of various sizes occluded most of the tubules. In Figure 5(c), with artificial saliva immersion, the dentine surface appeared to be covered by the layer, and there were different formations covering the tubules. After an acid challenge, the results were also encouraging as many of the dentine tubules were still occluded, and although the surface layer was dissolved away, there was evidence of tubular occlusion within the tubules (Figure 5(d)).

**Figure 6:**
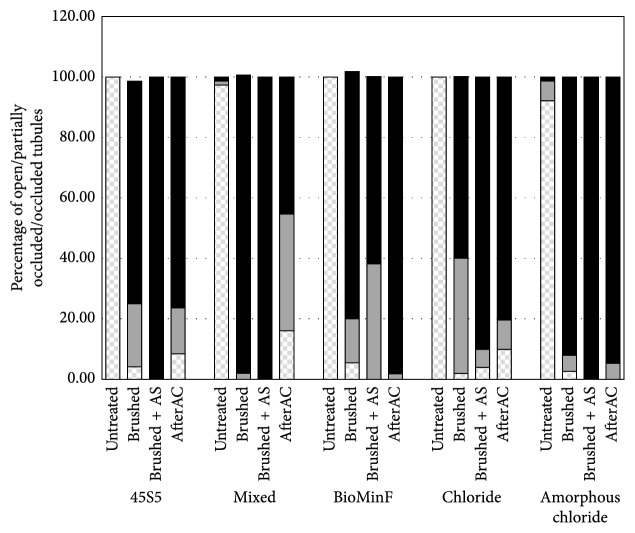
Percentage of open tubules (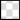), partially occluded tubules (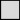), and occluded tubules (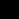) present after different treatments.

Figure 6 shows the percentages of open tubules, partially occluded tubules, and occluded tubules estimated from the SEM micrographs. It can be observed that all the toothpastes exhibited a significant increase in occlusion after brushing, with over >90% of all treatments resulting in complete and partially occluded tubules after brushing. Furthermore, the tubule occlusion generally increased upon immersion in AS but generally declined after the acid challenge in citric acid. The exception here was the BioMinF which shows almost 100% tubule occlusion after the acid challenge.

When comparing the control images with those images following brushing with the bioactive glass-based toothpaste formulations, it may be concluded that all samples provided a degree of tubule occlusion following brushing with the respective toothpastes (Figures 1–5).

## 4. Discussion

It was evident from previous in vitro studies that bioactive glasses have the capacity for occluding dentine tubules and as such incorporating these glasses into toothpaste formulations may be beneficial for those individuals who suffer from DH. In the present in vitro study, artificial saliva was formulated to simulate the in vivo environment following brushing the teeth. The results indicated that there was a good surface deposit with characteristic plate-like crystals and needle crystal formations in the different samples.

A study by Wang et al. [17] compared different types of bioactive glass-based toothpastes. Although these formulations were designed to deliver potassium ions to treat DH, the silica and calcium particles clearly occluded the tubules together with the smear layer produced by the application of the toothpaste. The SEM images of the toothpastes indicated that the toothpastes showed no resistance to an acid challenge, and the citric acid removed the particulate-coating layer from the tubule orifices. Furthermore, the precipitates formed were more resistant to an acid challenge when inside the dentine tubules. Another observation from this study which was of interest was that when a tooth was immersed in artificial saliva, there was an increase in fluid flow as measured in a hydraulic conductance model. This observation is somewhat contradictory as one would have expected a decrease in fluid flow following immersion.

In the 45S5 sample investigated in the present study, there was an indication that the Bioglass formulation does not form any apatite directly and may form an octacalcium phosphate (OCP) phase, which has a plate-like morphology. OCP is recognized to be a precursor for hydroxyapatite formation when the pH is ≤9 in the absence of fluoride. Moreover, the fluoride ions can either aid in the conversion of OCP to apatite or result in direct apatite formation. Therefore, OCP has an attractive potential for remineralization since OCP can incorporate a source of fluoride for catalyzing the transformation of OCP to apatite and for the formation of a more acid-durable fluoridated apatite [18].

In a novel laboratory study [15], the investigators synthesized a varnish containing potassium chloride (KCl) and fluoridated hydroxyapatite (FHA). The SEM images obtained from this study indicated that the dentine tubules were occluded in the varnish FHA group. The KCl-FHA varnish could release potassium ions and reduce hydraulic conductance of the dentine discs and may therefore be a suitable option for the treatment of DH. FHA has the ability to occlude dentine tubules over time; however, the samples were not subjected to an acidic challenge or any other food and beverages that would have an impact in the clinical environment.

In the present study, the SEM images demonstrated evidence of hydroxyapatite formation as several hexagonal crystals were observed within the images following artificial saliva immersion. Apatite has a hexagonal structure that can be easily recognized. Moreover, hydroxyapatite is relatively stable when compared to many other calcium phosphates, under physiological conditions although it is not as stable as fluorapatite. When exposed to an acid challenge, most of the layer formed with the artificial saliva, which is thought to be hydroxyapatite, was dissolved, although there was still evidence of some tubule occlusion. The BioMinF treatment resulted in a layer consisting of fine highly elongated needle-like crystals in contrast to the more plate-like crystals or short stubby crystals formed with the fluoride-free toothpastes. Fluoride is known to promote the formation of fluorapatite, which generally forms as needle-like crystals and is much more acid durable. The BioMinF treatment resulted in the greatest tubule occlusion following the acid treatment, which probably reflects the formation of a more acid-resistant fluorapatite.

The needle-like formations, similar to those observed in the images, were also observed in both the chloride glass and amorphous chloride glass images. A study by Iijima et al. elucidated that the formation of needle-like structures after immersion in artificial saliva was enriched with both Ca and P, and as such the bioactive glass-coated alumina produced a crystal which may be calcium phosphate [19, 20].

Recent studies have made considerable progress in elucidating the effects of Bioglass particles on the tooth structure through the in vitro evaluation of different chemical composition(s) of Bioglass-containing toothpastes [21]. For example, several studies have reported different outcomes when evaluating chloride-containing toothpastes. Several in vitro studies have demonstrated that a small crystalline deposit was precipitated onto the dentine surface which can easily be rinsed away; however, other studies have reported positive effects of the chloride-containing products on the relief of DH [15, 22].

When comparing chlorine and fluoride toothpaste formulations, there were conflicting results reported in the published literature with several clinical studies indicating that there were no differences in efficacy between the two products and other studies indicating that there were differences in favour of chloride-containing toothpastes. This observation was also true when comparing in vitro studies alone as well as comparative studies using cross sections of dentine where no tubular occlusion was observed following treatment with an SnF_2_-containing toothpaste [23].

A study published in 2013 [24] aimed at comparing the effectiveness of a one-minute application with a polishing prophylaxis paste containing 15% calcium sodium phosphosilicate with and without fluoride compared to a fluoride polishing control in reducing post-therapy DH following a dental scaling and root planning procedure. DH was assessed by both tactile and air blast stimuli at baseline, immediately following polishing and 28 days after the single application (subjects were provided with a non desensitizing toothpaste for the duration of the study). The results showed a significant reduction of sensitivity after 28 days of treatment for both groups with or without fluoride which would suggest that any improvement in DH was independent of the presence of fluoride (note that there are many confounding factors in running DH studies which could affect the results in this type of study) [25].

A further in vitro study [8] reported that the toothpaste formulations with different proportions of Bioglass replacing the silica compounds (2.5% and 7.5%) provided a greater surface coverage than the original Bioglass product. However, the study did not assess the composition of the particles deposited on the dentine surface or within the dentine tubules. Furthermore, there was no reported determination on whether the deposit was an abrasive component, for example, silica, or whether the deposit consisted of Bioglass particles or a precipitation of calcium phosphate following ion exchange on the surface of Bioglass.

Although the in vitro effectiveness of potassium-containing toothpastes was not the focus of the present study, a brief comparison of the effectiveness of products that occlude the dentine tubules (tubular occlusion) compared to the effect of potassium-containing toothpaste that claimed to work by blocking a pulp nerve response is worthy of some comment. For example, Acharya et al. compared the efficacy of toothpastes containing calcium sodium phosphosilicate and potassium nitrate and reported that calcium sodium phosphosilicate had a greater reduction in DH than potassium nitrate [11]. Moreover, this systematic review presented an overview on clinical trials of calcium sodium phosphosilicate (CSPS) to treat DH. CSPS was reported to provide superior results in reducing DH compared to potassium nitrate-containing toothpastes. Investigators have also suggested that when in contact with body fluids, CSPS reacts forming a layer of hydroxyapatite that can occlude dentine tubules. The results with CSPS were also reported to be more effective than the negative controls [26].

It is recognized, however, that the quantification of the number of occluded tubules is somewhat subjective, and perhaps, the limitation in the present study was the lack of fracture specimens to view the depth of penetration into the tubules. Nevertheless, the semi-quantification method used in the study does the support the observations from the SEMs that there was a degree of tubular occlusion which varied between the experimental toothpastes. Furthermore, evidence from an unpublished report that included this study indicated that the hydraulic conductance values of the BioMinF toothpaste supported the SEM observations indicating that the dentinal tubules were occluded [27].

One final review that may be relevant in this discussion on the effectiveness of CSPS is a systematic review by Talioti et al. [28]. These investigators compared the evidence of OTC desensitizing products (e.g., calcium sodium phosphosilicate, amorphous calcium phosphate, nanohydroxyapatite, and tooth mousse toothpaste/gels) in reducing DH. One of the problems reported by these investigators was that there was a lack of published studies directly comparing these four products. Furthermore, although there was evidence for the effectiveness of CSPS in occluding the dentine tubules from the in vitro studies, no conclusion could be made regarding the clinical efficacy of the various desensitizing toothpastes compared in the review. This was due in part to the different study designs and methodologies used in the various studies and the fact that there were relatively few randomized controlled trials (RCTs) available for analysis [28]. The systematic review and meta-analysis by Zhu et al. [26] also recognized the limitations of the published studies evaluating CSPS formulations in both toothpastes for DH and in prophylaxis polishing pastes for post periodontal therapy hypersensitivity. These investigators recommended that further non Industry (independent) supported clinical studies should be conducted prior to making any definitive recommendations regarding the efficacy of these formulations.

## 5. Conclusions

Of the various compositions of bioactive glasses assessed in the present in vitro study, all glass compositions demonstrated surface coverage after brushing with the formulated toothpaste. The formation of a hydroxyapatite layer occluding the dentine tubules following artificial saliva immersion may be considered an important stepping stone for further evaluation of these bioactive glass compositions. One of the innovative features of the study was the incorporation of an acid challenge to mimic the oral environment. Although the glass formulations, in particular Biomin, were resistant to an acid challenge, there was no doubt that further research was required to identify a different formulation or component that was not removed when immersed in a citric acid solution. In conclusion, the results from the present in vitro study would appear to support the growing evidence in the published literature that toothpaste formulations containing bioactive glasses occlude dentine tubules and therefore may be an effective approach treating DH.

## Figures and Tables

**Table 1:** Five different types of bioactive glass formulations used for the study.

(1)	Laboratory-manufactured 45S5
(2)	Mixed glass containing fluoride and chloride
(3)	Commercially available BioMinF
(4)	Chloride glass
(5)	Amorphous chloride glass

**Table 2:** Test and control discs with the specific method of application of the toothpaste formulations.

Section 1	Control
Section 2	Brushed with toothpaste
Section 3	Brushed with toothpaste + artificial saliva
Section 4	Brushed with toothpaste + acid challenge
